# Etiology of Dental Caries

**Published:** 1883-01

**Authors:** George Watt


### ARTICLE III.

### *The Etiology of Dental Decay.*

BY GEORGE WATT, M. D., D. D. S.,

[Read before the Ohio State Dental Society.]

For many centuries the profession rested in absolute quiet and content, holding that the well-known decay of the teeth resulted from inflammatory disease, regarding the term inflammatory in its ordinary acceptation without reaching out for such definitions as "retrograde metamorphosis of tissue,"—definitions intended to be broad enough to enwrap and hide from view the ignorance of the definer.

As long as the above views were universally held in reference to the etiology and pathology of dental decay, as indicated by the universal adoption of the term dental caries, no progress was made, or could be made, toward rational and proper treatment of this condition. I have elsewhere said, that the discovery and recognition of the fact that dental caries is something else than an ulcer, laid the foundation of dental surgery. This position was criticised with attempted severity by one who is diligently working to rool back the car of dental progress, and who, by virtue of an accidental position, apparently reached as an advertising dodge, may be able to accomplish something in his favorite direction, if still permitted to spend his strength where true progress has not yet reached its average momentum.

For more than 30 years, as some of you can testify, I have endeavored to impress on my professional brethern that this is altogether the most important question in dental science; and daily I am pained to see how little progress has been made in the third of a century referred to. So little clear thought is yet manifested, that even to-day, not only with the less educated part of our profession, but often with advanced thinkers, it is not thought necessary, or even important, to recognize varieties in dental decay.

Though the three principal varieties are not more alike than a horse, a cow and a sheep,—not more alike than boils, tetter and chilblains—they are spoken of as identical. Phenomena are referred to as found in a carious tooth without the slightest hint as to which variety of caries is spoken of. And this is as true of men claiming to be eminent in our profession as it is of the tyros.

It is well known that the physical phenomena of dental caries are not greatly varied. But three, or if we include what is sometimes called "chemical abrasion," four distinct varieties are found, and the various modifications and combinations of these will explain all the recognized different appearances.

Many of the phenomena of caries can be observed as well, or better, by the unaided eye as by magnifiers. The black, brown, yellow, orange and white colors illustrate this. And the difference in texture of the different varieties is as observable without glasses as with them. No one can fail to see, in the most common form of decay, a state similar to what is sometimes called gelatinous bone, where, to show the organic structure, the solid portions of the bone are dissolved out by hydrochloric acid. The unaided eye can see that, to a good degree, the solid materials are gone from the softer textures; and it can recognize a decided difference in the textures of black and white decays,—can see a complete breaking down in the latter, with but little displacement of materials in the other. Yet at the present time quite a number of writers for the periodicals ignore all these differences, even though they talk much of the microscope, learnedly of leptothrix, and abound in bacteria and other bugs and bushes; and with dogmatic assertion, try to carry us back to the principles held before the dawn of dental surgery—principles which had to be discarded in order that dentistry migh spring into existence and take rank as a profession.

Let it not be supposed that I undervalue microscopic research. Far from it. I gave the last dollar I had, to purchase a better microscope than any I know of in the West, and borrowed money to pay my way home, even though I did not expect to have either time or opportunity to use the instrument in prolonged research. But let me remark that far too much confidence is placed in the statements of those who observe with high powers. This is evident from the almost constant contradictions of observers. There is great danger of optical delusions when observing with very high powers; and the danger increases in proportion to the squares of the powers. Hence the higher the power necessary to make any observation, the more caution is called for in receiving the testimony thus elicited. I am more than delighted to see these researches, but it is desirable to get the good without the evil from them. It is not strange that they receive too much credence. Human faith naturally takes hold on mystery. It is analogous to the weight given to chemical analysis in the popular mind, and which often results in the hanging of innocent men.

The germ theory of decay is lately pressed with considerable zeal. In all articles I have read in this direction, the writers show a very decided lack of clear thought as to what is held by the advocates of the so-called chemical theory. Some of them display absolute ignorance in this direction. But this is not strange, for even some holding to the "acid theory" have almost equally clouded views.

Now and then, through all the years of my connection with the dental profession, some wiseacre gets up to tell us that some constitutions are so defective that the dental organs are not well developed, and therefore decay easily. And even when they are all that ought to be expected from nature, by disease, constitutional or local, the buccal fluids become vitiated and cause the teeth to dacay; and then he tells us that therefore the chemical theory does not explain all, and he announces his discovery of the "chemicovital theory," and feels that he has lifted the whole profession out of the slough of ignorance.

Of course you all know that the most enthusiastic advocate of the chemical, or acid theory recognizes all these constitutional or local predispositions, but he holds at the same time, that no tooth is so badly developed that it goes into decay without an exciting agent.

To illustrate what has been termed a lack of clear thought in reference to the chemical theory of decay, it may be necessary to make a few somewhat lengthy quotations. It is hoped the reader or listener will be patient in proportion to the importance of the subject.

In a paper read by Prof. C. W. Spalding, at the annual meeting of the Illinois State Dental Society for 1881, he says: "In common, I believe, with many other practitioners, I have supposed that an acid condition of saliva prevails, or at least may usually be looked for in most cases of rapid decay of the teeth."

Now, while all the above is true, it shows great lack of clear thinking in reference to the acid theory of decay, which the Professor seems to countenance mainly in this article. Acids diffused throughout the buccal fluids, and therefore quiescent, can not cause dental caries. If the proper acid, and sufficiently concentrated, it may corrode the teeth, especially if dentine or cementum is exposed. But the acid which excites caries, as Dr. W. A. Pease lately expressed it, is generated, "*de novo*, on the spot."

Professor S. goes on to say: "My first experiments were made upon the saliva of a young girl (aged 12), whose teeth were decaying rapidly, so much so that fillings previously inserted, that is before the case came into my hands, whether of gold or other materials, had lasted but a short time, and in whose teeth new cavities were constantly forming, and this at so rapid a rate that cavities of considerable size would form in the space of a few months, notwithstanding pretty thorough cleanliness and good general care. I looked to find an acid reaction of the saliva in this case, and had been reflecting on a course of medical treatment, having in view the correction of the supposed condition of the saliva. What, then, was my surprise, on finding, after repeated tests, that the saliva of this young person exhibited in every test either a neutral or a slightly alkaline chemical reaction! In no one of a large number of tests was there any, even the smallest acid reaction shown. I immediately sought other cases where a similar destructive process was going on, but the result in each case was precisely the same as in the case just narrated. * * *

A professional friend also made similar tests in some very marked cases of rapid decay, with the same results—no acid condition of saliva revealed."

It is unfortunate that Prof. S. and his friend have failed to tell us what variety of decay prevailed in these cases. By making this omission they have almost failed to tell us anything about them; but it is quite probable the decay was light colored. It is well known that black decay is the least destructive and the slowest in progress of the several varieties of caries, while the light colored is the most rapid and disastrous. It is well recognized, too, by the thoughtful, that an alkaline state of the buccal fluids is a usual precursor of white decay. Many of you will bear me witness, that in the college, and in this society and others, I have diligently warned you of the danger of an alkaline reaction of the saliva, and have persistently urged an acid course of constitutional treatment whenever white decay is prevalent, or tartar is abundantly deposited. I have talked about pickles, vinegar, lemonade, saur-kraut, etc., till your ears have tingled with the names. And why? All well-posted advocates of the "chemical theory of decay believe that the exciting agent—the immediate thing that does the business, without anything between it and the mischief—acts in its nascent state' results from two distinct processes, the effect, that is, the nascent agent's activity, being the same. It may be produced either by synthesis or analysis, as for example, if sulphur is oxidized in the presence of water, sulphuric acid results, and thus resulting, it is nascent. Or a sulphate may be decomposed, the base combining with some other acid, and thus setting the sulphuric acid free, and in this condition it is nascent. You all know that in their nascent state chemical re-agents manifest greatly increased powers.

[TO BE CONTINUED.]

